# Green Hydrothermal Synthesis of Mn_3_O_4_ Nano-Octahedra Using Carménère Grape Pomace Extract and Evaluation of Their Properties for Energy Storage and Electrocatalysis

**DOI:** 10.3390/nano15161282

**Published:** 2025-08-20

**Authors:** Javier Lorca-Ponce, Paula Valenzuela-Bustamante, Paula Cornejo Retamales, Nicolas Nolan Mella, Valentina Cavieres Ríos, María J. Pérez Velez, Andrés M. Ramírez Ramírez, Leslie Diaz Jalaff

**Affiliations:** 1 Centro de Excelencia en Materiales Avanzados-Nanotecnología, LEITAT Chile, Santiago 7500724, Chile; javier.lorca@usach.cl (J.L.-P.); pvalenzuela@leitat.cl (P.V.-B.); pcornejo@leitat.cl (P.C.R.); nnolan@kolmenares.cl (N.N.M.); vacavieres@alumni.uc.cl (V.C.R.); 2 Departamento de Química, Facultad de Ciencias, Universidad de Chile, Santiago 7800003, Chile; mari.perez@alumnos.uai.cl

**Keywords:** hydrothermal, green synthesis, nano-octahedra, *Mn*_3_ *O*_4_, electrochemical properties, Carménère pomace

## Abstract

In this study, a green hydrothermal synthesis method was employed to produce *Mn*_3_*O*_4_ and *Mn*_3_*O*_4_/β-MnO_2_ nanostructures using EET-50, an organic extract obtained from a by-product of Carménère wine production. The biomolecules in EET-50 acted as reducing agents due to their electron-donating functional groups, enabling nanostructure formation without the need for additional chemical reductants. Morphological characterization by SEM revealed that a KMnO_4_/EET-50 mass ratio of 3:1 led to the synthesis of nano-octahedra alongside rod-like structures, with shorter reaction times favoring the development of isolated nano-octahedra ranging from 100 nm to 170 nm. Structural analyses by XRD and Raman spectroscopy confirmed the formation of mixed-phase *Mn*_3_*O*_4_/β-MnO_2_ and *Mn*_3_*O*_4_ (hausmannite). Electrochemical performance tests demonstrated that *Mn*_3_*O*_4_ nano-octahedra exhibited a superior specific capacitance of 236.27 F/g at 1 mA/g, surpassing the mixed-phase sample by 28.3%, and showed excellent capacitance retention (99.98%) after 100 cycles at 8 mA/g. Additionally, the *Mn*_3_*O*_4_ nano-octahedra exhibited enhanced oxygen evolution reaction performance in alkaline media, with an overpotential of 0.430 V vs. RHE and a Tafel slope of 205 mV/dec. These results underscore the potential of *Mn*_3_*O*_4_ nano-octahedra, synthesized via a green route using grape pomace extract as a reducing agent, offering an environmentally friendly alternative for applications in energy storage and electrocatalysis.

## 1. Introduction

Nanostructured materials have garnered significant interest in recent years due to their exceptional properties and wide range of applications compared to bulk materials [1,2,3]. Among these, manganese oxide nanostructures (Mn_x_O_y_) have received growing attention due to their notable characteristics, including their natural abundance, low production cost relative to noble metals, excellent biocompatibility, high chemical stability, corrosion resistance, and high specific capacitance [4]. Various research groups have focused on the synthesis and characterization of nanostructured manganese oxides, due to their potential applications in medicine, biosensors, wastewater treatment, and as electrochemical energy storage and electrocatalysis, particularly in oxygen reduction and evolution reactions (ORR/OER) [2,5,6].

Among manganese oxides, nanostructured *Mn*_3_*O*_4_ has attracted considerable attention due to its promising applications in sensors, photocatalysis, ion exchange, batteries, and supercapacitors [5,7,8,9]. In this context, hausmannite—the mineralogical form of *Mn*_3_*O*_4_ with tetragonal spinel structure— has been extensively studied as an electrode material owing to its outstanding characteristics, including high theoretical capacitance (1370 F/g), variable oxidation states, wide potential window, environmental friendliness, and cost-effectiveness [10,11,12]. Significant efforts have been made to explore and enhance the electrochemical performance of this material [13].

The spinel structure of *Mn*_3_*O*_4_ consists of a unit cell with 32 oxygen atoms and 24 manganese cations, both divalent and trivalent. Its remarkable physicochemical properties arise from its unique structural features: (i) cubic packing of oxide ions, (ii) tetrahedral coordination of Mn^+2^, (iii) octahedral coordination of Mn^+3^, and (iv) Jahn–Teller distortion induced by high-spin d^4^ Mn^+3^ atoms. *Mn*_3_*O*_4_ nano-octahedra, composed of eight (111) crystal planes, exhibit particularly interesting properties. Consequently, extensive research has been devoted to these nanostructures, with a focus on enhancing performance through the synthesis of monodisperse *Mn*_3_*O*_4_ nanocrystals with tailored morphologies, sizes, and specific surface areas [10,11,12].

Among the polymorphs of MnO_2_, β-MnO_2_ has received considerable attention due to its excellent electrochemical stability, tunnel-type crystalline structure, and high theoretical capacity, making it suitable for applications in catalysis, sensors, batteries, and supercapacitors [14,15]. The formation of *Mn*_3_*O*_4_/β-MnO_2_ heterostructures can synergistically combine the high electrical conductivity and fast redox kinetics of *Mn*_3_*O*_4_ with the structural stability and catalytic activity of β-MnO_2_ [16]. Despite their potential, the green synthesis of such heterostructures remains largely unexplored, particularly using natural reducing agents derived from agro-industrial by-products.

Several synthetic approaches have been explored to obtain nanostructures, including chemical methods such as sol-gel, thermolysis, hot injection, and hydrothermal synthesis [17,18,19,20,21]. However, conventional chemical routes typically require toxic reducing agents such as nitric acid, sodium hydroxide, hydrochloric acid, sodium borohydride, hydrazine, and sodium citrate, among others [22,23,24], which pose significant environmental and health risks [25,26]. Therefore, due to their simple, environmentally friendly manufacturing process, low cost, and high product purity, green and biological synthesis offer fundamental advantages [27].

Plant-derived components contain phytochemical compounds that are essential for the synthesis of manganese oxide nanostructures. In addition to performing functions such as reducing protective and stabilizing agents for nanostructures, they also prevent the agglomeration of the nanostructures during the growth of the material. Phytochemicals present in various parts of plants (leaves, fruits, stems, peels, seeds, and flowers) are a rich source of biological reducing agents [4]. Various studies have identified a variety of natural compounds in plant extracts, such as polyphenols, flavonoids, and terpenoids, among others, which participate in the reduction of manganese precursor salts to metal oxide nanostructures and contribute to their stability in redox processes mediated by the formation of complexes [26]. Specific research on the green synthesis of nanostructured manganese oxides has revealed the presence of phytochemicals, such as the flavonoid epigallocatechin gallate (EGCG), in green tea leaf extracts [26], as well as polyphenols and flavonoids in cinnamon extracts [28]. In addition, compounds such as saponins have been identified in the leaves of *Fagonia cretica* [29], citric acid and ascorbic acid (vitamin C) in lemon juice, and flavonoid glucoside, coumaric acid, β- and γ-sitosterol, as well as volatile oils in citrus peel [30].

In this work, the green hydrothermal synthesis of *Mn*_3_*O*_4_ nano-octahedra using a grape pomace extract obtained from the Carménère variety as a reducing agent is reported. The effects of reaction time and precursor-to-extract ratio on the resulting nanostructure morphology were investigated, and the possible formation mechanisms were discussed. The resulting manganese oxide nanostructures were evaluated for their capacitive behavior and charge–discharge cycling performance in energy storage applications, as well as for their electrocatalytic activity in the oxygen evolution reaction (OER) under acidic, neutral, and alkaline conditions.

## 2. Materials and Methods

### 2.1. Materials

The grape pomace (*Vitis vinifera* L.) used in this study corresponded to the Carménère variety that was donated by Viña Concha y Toro during the 2022 harvest in the Region del Maule, Chile (35°25′36″ S 71°40′18″ W).

The Folin–Ciocalteu’s phenol reagent, gallic acid (C_6_H_2_(OH)_3_COOH, phyproof^®^ Reference Substance >98% HPLC), sodium acetate (CH_3_COONa, EMSURE^®^ ACS), potassium hydroxide (KOH, ACS), sulfuric acid (H_2_SO_4_, ACS) and sodium sulfate (Na_2_SO_4_, ACS >99%) were purchased from Merck (Darmstadt, Germany). 6-Hydroxy-2,5,7,8-tetramethylchroman-2-carboxylic acid (Trolox, >98%), aluminum chloride (AlCl_3_, ReagentPlus^®^, 99%), iron(III) chloride hexahydrate (FeCl_3_·6H_2_O, EMSURE^®^ ACS), triphenyl tetrazolium chloride (TPTZ, ≥98%) and 2-diphenyl-1-picrylhydrazyl (DPPH) from Sigma-Aldrich (St. Louis, MO, USA); potassium permanganate (KMnO_4_, EMSURE^®^ ACS) was acquired Sigma-Aldrich (Burlington, MA, USA). Sodium carbonate (Na_2_CO_3_, p.a. EMSURE^®^ ISO), ethanol (CH_3_CH_2_OH, 96%) and methanol (CH_3_OH, EMSURE^®^ ACS) by Supelco (Darmstadt, Germany). All the chemicals were used as received. MilliQ water with a conductivity of 0.055 μS/cm at 25 °C was used (Millipore, Burlington, MA, USA filter system, Millipore Co.).

### 2.2. Method of Obtaining Grape Pomace Extract

The pomace is a solid residue recovered after the pressing process (in this case, from the Carménère grape variety) and the alcoholic fermentation of the wine, and it consists of skins, seeds, and a small percentage of stems. The samples were packed in dark polyethylene bags and immediately frozen at −20 °C.

Prior to extraction, most of the large stems were manually removed, and the pomace was ground to obtain a uniform plant material particle size. Then, a determined amount of frozen and ground pomace was used for the extraction of phenolic compounds. This extraction was performed through dynamic maceration, following a previously reported methodology, described below [31].

A 50% *v*/*v* hydroalcoholic mixture was prepared and used as the extraction solvent. The pomace was mixed with the solvent at a 1:4 (*w*/*v*) ratio, and the suspension was stirred at 250 rpm for 90 min at 50 °C in a beaker sealed with aluminum foil. The procedure was repeated once under the same conditions. Finally, both extracts were combined, and the solvent was removed by evaporation in a rotary evaporator (Hei-VAO Advantage, Heidolph Instruments GmbH & Co. KG, Schwabach, Germany). The concentrated extract was then freeze-dried, and the resulting powder was stored in an amber glass container at 8 °C under the label EET-50.

### 2.3. Characterization of Carménère Grape Pomace Extract

Total phenolic content (TPC) was determined by the Folin–Ciocalteu method [31]. Briefly, 100 µL of the EET-50 diluted in ethanol 40% *v*/*v* was mixed with 100 µL of the Folin–Ciocalteu reagent (diluted at 10% *v*/*v*). This mixture was incubated at 40 °C for 2 min, after which 800 µL of 5% *w*/*v* Na_2_CO_3_ was added. Subsequently, the solution was incubated for 20 min at 40 °C. Absorbance was measured at 760 nm, and the results were expressed in mg of gallic acid equivalents per gram of dry weight extract (mg GAE/g dw).

Additionally, total flavonoid content (TFC) was determined by a colorimetric method, as described by Peña-Cerda et al. [32]. In brief, 100 µL of EET-50 dissolved in 80% *v*/*v* ethanol was mixed with 20 µL of a 10% *w*/*v* AlCl_3_ solution, 20 µL of 1M CH_3_COONa, and 560 µL of Mill-Q water. The mixture was incubated for 40 min at 25 °C, and absorbance was measured at 415 nm. Results were expressed in mg of quercetin equivalents per gram of dry weight extract (mg QE/g dw).

Total monomeric anthocyanin content (TMA) was determined according to the method described by Lee et al. [33] The colored flavylium cation predominates at pH 1.0, while the colorless hemiketal form is dominant at pH 4.5. To quantify TMA, the difference in absorbance between the samples at the two pH levels was calculated using Equation (1):

(1) $$A={\left(A_{520nm}-A_{700nm}\right)}_{pH1.0}-{\left(A_{520nm}-A_{700nm}\right)}_{pH4.5}$$

Then, the TMA concentration was obtained by the following calculation (Equation (2):

(2) $$Total\;Monomeric\;Anthocyanin\;\left(mg/L\right)=\;(A\times MW\;\times DF\times 1000)/(\varepsilon \times 1)$$

*A* corresponds to the absorbance at different pH, *MW* is the molecular weight, *DF* is the dilution factor, *ε* is the molar absorptivity derived from a full wavelength scan, and 1 is for the path length (in cm). The results were expressed in mg of cyanidin-3-glucoside equivalents per gram of dry weight extract (mg CE/g dw).

### 2.4. Determination of the Antioxidant Activity of EET-50

The antioxidant activity of the Carménère grape pomace extract was evaluated by the following methods:

#### 2.4.1. FRAP Assay

The reduction of ferric-2,4,6, tripyridyl-s-triazine (TPTZ) complex was measured, wherein ferric iron (Fe^3+^-TPTZ) was reduced to ferrous ion (Fe^2+^-TPTZ) under acidic conditions, forming a blue-colored complex with an absorbance peak at 593 nm. The assay was performed according to the methodology reported by Benzie and Strain [34]. Briefly, 100 µL of EET-50 dissolved in methanol was combined with 2900 µL of FRAP reagent. The mixture was incubated at 37 °C for 60 min, and the absorbance was measured at 593 nm. The results were expressed in mmol Trolox equivalents per gram of dry weight extract (mmol TE/g dw).

#### 2.4.2. DPPH Radical Method

This assay was conducted according to the procedure described by Gonzales et al. [35]. A fresh DPPH solution was prepared in methanol at a concentration of 0.1 mM. A volume of 3 mL of this solution was mixed with 1 mL of EET-50, also dissolved in methanol. The mixture was incubated in the dark at room temperature for 30 min, and absorbance was measured at 517 nm. The control consisted of DPPH solution mixed with 1 mL of methanol, and its absorbance was used in Equation (3) to calculate the percentage inhibition of EET-50 as follows:

(3) $$DPPH\;Inhibition\;\left(\%\right)=\frac{\left(Abs\;Control-Abs\;Extract\right)}{Abs\;Control}\times 100$$

Different concentrations of EET-50 were evaluated to obtain a % inhibition vs. concentration curve. The Inhibitory Concentration 50 (IC_50_, µg mL^−1^) of the extract was calculated by logarithmic adjustment using the GraphPad Prism program version 10.0.0. All samples were evaluated in triplicate.

### 2.5. Synthesis of Mn_3_O_4_ and Mn_3_O_4_/β-MnO_2_

Aqueous solutions of 3% *m*/*v* KMnO_4_ and 1% *m*/*v* EET-50 were prepared and homogenized separately in two flasks. The EET-50 solution was then added dropwise to the KMnO_4_ solution using a syringe fitted with a 0.22 µm filter (Sigma-Aldrich, Burlington, MA, USA) to remove any suspended solids. The addition rate was maintained at 1 drop per second. The resulting mixture was stirred at 300 rpm for 1 h at room temperature before the hydrothermal treatment to ensure homogeneous distribution of the reactants and to allow a controlled pre-reaction between KMnO_4_ and EET-50.

Subsequently, the solution was transferred to a 25 mL Teflon-lined stainless steel autoclave reactor and heat-treated in a preheated lab oven Memmert V029 at 180 °C for 3 h. Upon cooling to room temperature, a brown precipitate was observed. The solid product was filtered by vacuum filtration using a Lab Tech VP50 Plus vacuum pump and Durapore^®^ membrane filters (type 0.65 µm DVPP), washed with ethanol, and air-dried at 60 °C for 24 h. Finally, the dried material was calcined in a Carbolite Gero CWF 12/13 muffle furnace at 300 °C for 3 h using a heating ramp of 20 °C/min to obtain *Mn*_3_*O*_4_ nanostructures. After the hydrothermal treatment, the autoclave was allowed to cool naturally to room temperature.

To synthesize the *Mn*_3_*O*_4_/β-MnO_2_ (mixture of nano-octahedral and nanorods structures), the same procedure was followed, except the reaction time in the Teflon-lined stainless steel autoclave reactor was extended to 12 h. This modification led to the formation of a product with a distinct phase composition.

### 2.6. Characterization of Nanostructures

The morphology of coated and uncoated substrates was examined using a field emission scanning electron microscope (Inspect F50 FEI, Thermo Fisher, Waltham, MA, USA) operated at an accelerating voltage of 20 kV. Each specimen was mounted on an aluminum holder and coated with a 10 nm gold layer using a TEDPELLA 108 sputter coater coupled to an MTM 20 Cressington thickness controller.

The crystalline structure of the products was analyzed by X-ray diffraction (XRD) using a D8 Advance diffractometer (Bruker, Billerica, MA, USA) with monochromatic Cu Kα radiation (λ = 1.5405 Å). The 2θ range was set from 10° to 80°, with a step size of 0.02° and a scan rate of 0.3 s per step.

Raman spectra were recorded using a B&W Tek i-Raman Plus 532 (Metrohm, Herisau, Switzerland), coupled with and Raman Video Microsampling System, equipped with a 532 nm laser over the spectral range of 0 to 3000 cm^−1^.

### 2.7. Electrochemical Analysis 

Electrochemical measurements were performed using a CH Instruments potentiostat/galvanostat CHI900B (CH Instrument, Bee Cave, TX, USA) in a single-compartment cell with a three-electrode configuration. A platinum wire was used as the counter electrode, an Ag/AgCl (0.197 V vs. RHE) as the reference electrode, and a glassy carbon (GC) as the working electrode. The GC electrode was modified with three sequential applications of 5 µL (15 µL total) of a 1 mg/mL dispersion of the electroactive material, with each layer dried for 15 min under an infrared lamp at ambient conditions.

Capacitance studies were conducted in 1 M Na_2_SO_4_ using cyclic voltammetry within a potential range of −0.2 V to 0.8 V at different scan rates (0.01–0.150 V/s). Charge–discharge process at constant current (CDG) tests were performed at various current densities from 1.00 to 16.00 mA/g.

The catalytic activity towards the oxygen evolution reaction (OER) was evaluated on acidic (0.5M H_2_SO_4_), neutral (1M Na_2_SO_4_), and alkaline (1M KOH) conditions using linear sweep voltammetry (LSV) over a potential range of 0.8 V to 2.2 V at a scan rate of 0.002 V/s.

All electrochemical measurements were performed at 20 °C under nitrogen atmosphere to ensure inert conditions.

### 2.8. Capacitance Estimation

The specific capacitance (C) of the electrodes modified with *Mn*_3_*O*_4_ and *Mn*_3_*O*_4_/β-MnO_2_ was calculated from cyclic voltammetry using Equation (4) [36]

(4) $$C=\frac{\int_{E_{1}}^{E_{2}}i\;\left(E\right)d(E)}{2vm(E_{2}-E_{1})}$$

where the integral, $\int_{E_{1}}^{E_{2}}i\;\left(E\right)d(E)$ represents the total charge, *E*_2_ and *E*_1_ are the inversion potentials of the cyclic voltammetry, *ν* is the scan rate, and *m* is the mass of active material deposited on the electrode, calculated from the aliquots applied.

The specific capacitance from the galvanostatic charge–discharge (GCD) measurements was determined using Equation (5) [37]

(5) $$C=\frac{it}{m\Delta V}$$

where *i* is the applied discharge current, *t* is the discharge time, *ΔV* is the potential window during the discharge process, and *m* is the same mass of active material used in Equation (4).

## 3. Results

### 3.1. Chemical Characterization and Antioxidant Activity of the EET-50

Grape pomace is considered an inexpensive source of high-value antioxidant phenolic compounds, including anthocyanins, flavan-3-ols, stilbenes, and phenolic acids [38]. Various ethanol concentrations were tested, with the 50% ethanol extract (EET-50) yielding the highest total polyphenol content (TPC) and antioxidant activity (Table 1).

Specifically, EET-50 achieved a yield of 7.8 ± 0.1% and a TPC of 149.9 ± 0.4 mg GAE/g dw, a value significantly higher than the TPC reported for extracts from grape pomace of different varieties. Caldas et al. [39] reported TPC values ranging between 5.7 and 48.6 mg GAE/g dw for ethanolic extracts of red sparkling wine pomace obtained through mechanical agitation. Similarly, Pintać et al. [40] extracted polyphenols from different grape pomace varieties using various solvents, reporting TPC values of 68.8 ± 0.04 and 65.2 ± 0.35 mg GAE/g dw for Cabernet Sauvignon and Merlot, respectively, using 80% ethanol. These extracts exhibit TFC values of 6.78 ± 0.30 and 6.11 ± 0.04 mg QE/g dw—approximately half the TFC observed in EET-50 (12.1 ± 0.9 mg QE/g dw). Flavonoids identified in Carménère grape pomace include quercetin, kaempferol, and rutin, along with compounds from the flavan-3-ol family, such as epicatechin and procyanidin [41].

The total monomeric anthocyanins (TMAs), a class of pigmented phenolic compounds found in high concentrations in both red wine and pomace, were also quantified. EET-50 contained 12.1 ± 0.4 mg CE/g dw of TMAs, a value comparable to its TFC. Since comparisons were made with studies employing conventional extraction via mechanical agitation, the higher TPC and TFC in EET-50 may be attributed to the use of a different grape pomace variety—in our case, Carménère—as well as differences in the type of solvent used for extraction. The extraction conditions—specifically solvent composition, temperature, and extraction time—are critical parameters that influence not only the efficiency of phenolic compound recovery but also the preservation of their chemical integrity and qualitative profile. In this study, a 50% *v*/*v* hydroalcoholic solution and an extraction temperature of 50 °C for 90 min were selected based on optimized protocols for polyphenol extraction, as ethanol concentrations between 50–70% have been shown to yield extracts with high antioxidant capacity [40,42].

Hydroalcoholic mixtures are widely used due to their ability to extract a broad range of phenolics with varying polarity, including flavonoids and anthocyanins. Moderate extraction temperatures (40–60 °C) enhance diffusion and cell permeability while preserving thermolabile compounds. In contrast, prolonged extraction times or elevated temperatures may cause hydrolysis of glycosylated flavonoids and oxidation of anthocyanins, ultimately reducing antioxidant potential [42].

Few studies report the chemical composition of Carménère grape pomace, making direct comparison challenging. Carménère is one of Chile’s signature wines, with an annual production of approximately 95 million liters, generating close to 22 million kilograms of pomace. Huamán-Castilla et al. [41] reported TPC values below 20.21 mg GAE/g dw for Carménère grape pomace extracts obtained via hot pressurized liquid extraction (HPLE)—approximately seven times lower than the TPC of EET-50 obtained by conventional extraction in this study.

The antioxidant activity of EET-50 was assessed using two complementary assays. The FRAP assay, based on a single electron transfer (SET), measures the reduction of ferric ions (Fe^+3^) to ferrous (Fe^+2^) ions, forming an intensely blue ferrous complex under acidic conditions. In contrast, the DPPH assay involves SET and hydrogen atom transfer (HAT) mechanisms [43]. EET-50 exhibited a FRAP value of 1.59 ± 0.04 mmol TE/g dw and an IC_50_ of 17.96 ± 0.87 μg/mL in the DPPH assay, significantly lower than the IC_50_ values (23.78 to 69.20 μg/mL) reported by Huaman-Castilla et al. [41] for ethanol extracts of Carménère pomace.

The antioxidant activity of EET-50 can be primarily attributed to its high content of phenolic compounds—particularly flavonoids and anthocyanins—along with other polar constituents encompassed within the total phenolic content. Although individual phenolics were not identified in this study, previous work has reported the presence of gallic, caffeic, and chlorogenic acids in Carménère pomace, albeit at lower concentrations than flavanols and anthocyanins [41]. These bioactive compounds were efficiently extracted using the selected hydroalcoholic conditions, which preserved their structural integrity. In addition to their DPPH radical-scavenging ability, EET-50 demonstrated strong ferric-reducing activity in the FRAP assay, reflecting the electron-donating capacity of polyphenols. Their hydroxyl and carbonyl groups enable electron transfer to metal ions, forming stabilized keto-enol structures [44]. Altogether, these results confirm that the EET-50 possesses a robust antioxidant profile, supported by both its chemical composition and redox behavior in vitro.

### 3.2. Synthesis and Characterization of the Nanostructures

#### 3.2.1. Scanning Electron Microscopy

Once the EET-50 extract was obtained and characterized, it was used as a reducing agent for the synthesis of manganese oxide nanostructures, following the procedure described in 2.5. In this section, the morphological evolution of materials is first presented as a function of KMnO_4_: EET-50 mass ratio. For these experiments, the reaction time and temperature were kept constant at 12 h and 180 °C, respectively.

**Figure 1:**
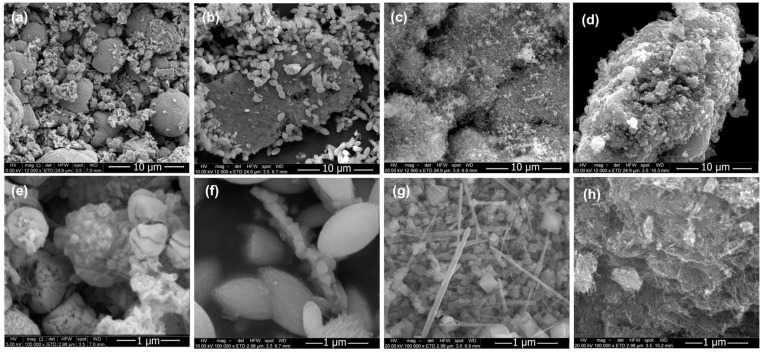
Morphology evolution process with KMnO_4_/EET50 (**a**,**e**) 1:2, (**b**,**f**) 1:1, (**c**,**g**) 3:1, and (**d**,**h**) 6:1.

Figure 1 presents SEM images at 12,000× and 100,000× for samples prepared with mass ratios of 1:2, 1:1, 3:1, and 6:1. In the samples prepared with a 1:2 ratio (Figure 1a), two distinct types of structures were identified: large spheres approximately 4–6 µm in diameter and smaller, irregular, rough structures of varying sizes. Higher magnification images for this ratio (Figure 1e) did not reveal the formation of octahedral structures.

In contrast, the sample prepared with 1:1 (Figure 1b,f) displayed three morphological features: a large agglomeration of 22.7 µm, various ovoid particles with an average size of 1.24 µm, and small aggregates with pyramidal or octahedral morphology. These findings suggest that increasing the KMnO_4_ concentration promotes structural diversity and the formation of a small fraction of nanostructured materials.

For the sample synthesized with a 3:1 ratio (Figure 1c,g), well-defined octahedral structures were predominant, with sizes ranging from 150–250 nm. These nanostructures were accompanied by bar-shaped particles of variable lengths. The prevalence of uniform nano-octahedra at this ratio indicates an optimal balance between precursor and reducing agent.

Finally, Figure 1d depicts the sample prepared with a 6:1 ratio, where a large structure measuring 30.3 µm was observed. When the surface of the aggregates was magnified (Figure 1h), the large structures appeared to be covered with thin needles, along with some aggregates with undefined shapes. No nano-octahedra structures were detected for this sample.

In conclusion, the KMnO_4_:EET50 mass ratio of 3:1 resulted in the highest yield of uniform nano-octahedral manganese oxide structures, demonstrating the importance of the precursor-to-extract balance in directing the morphology of the final product.

In a second experiment, morphological evolution was studied as a function of reaction time, while maintaining a constant KMnO_4_: EET-50 mass ratio of 3:1 and a temperature of 180 °C.

**Figure 2:**
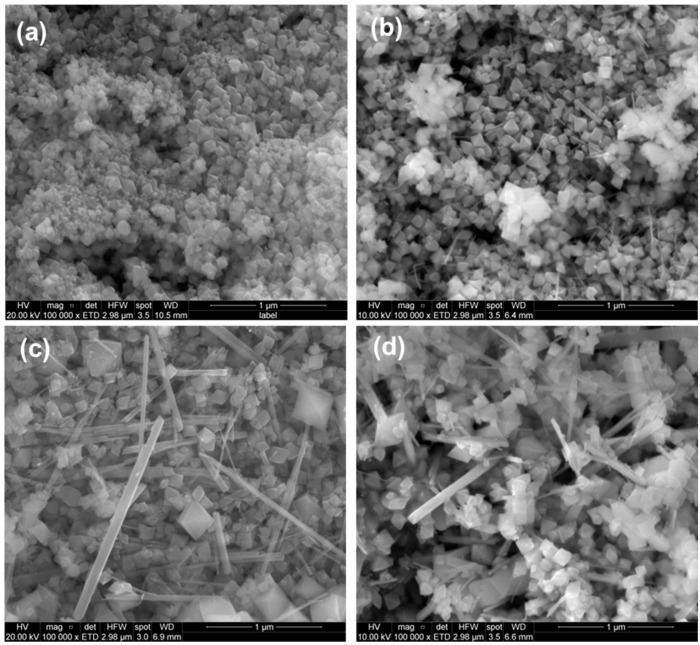
Morphology evolution process with the reaction time (**a**) 3, (**b**) 6, (**c**) 12, and (**d**) 18 h.

SEM images of the samples with reaction times of 3, 6, 12, and 18 h are presented in Figure 2. The sample with the shortest reaction time (Figure 2a) predominantly exhibited nano-octahedral structures measuring approximately 100–170 nm, with no evidence of rod-or needle-like formations. In contrast, Figure 2b shows that the sample reacted for 6 h developed octahedral structures similar in size and morphology to those in the 3 h sample, but with the additional presence of thin, strip-like formations.

Finally, the samples with reaction times of 12 and 18 h showed no significant differences, both featuring a mixture of rods and octahedra of varying sizes. This experiment demonstrates that shorter reaction times favor the formation of well-defined, isolated nano-octahedral structures.

To clarify the size and visualization of the obtained structures, high-resolution TEM images were provided in Figure S1 of the Supporting Information. These images offer a clearer view of the isolated octahedral structures as well as one of the rod-like formations present in the sample obtained after 12 h of reaction. Hydrothermal reaction time has proven to be a critical parameter in controlling both the crystalline phase and the morphology of manganese oxide structures.

To specifically evaluate the influence of the reaction temperature, additional experiments were carried out under the same conditions used to obtain the mixed-phase product, but at 80 °C. TEM images of the resulting materials were provided in Figure S2 of the Supplementary Information. These images do not reveal evidence of octahedral structures; instead, the material appears as aggregates with irregular morphologies and a few small needle structures. These results suggest that higher temperatures were required to drive the phase transition necessary for the formation of well-defined octahedral morphologies.

The results presented in this section can be correlated with previously published studies on hydrothermal manganese oxide synthesis. For example, Ashoka et al. [45] reported the formation of octahedral *Mn*_3_*O*_4_ via a homogeneous reduction route involving two distinct stages. Initially, MnOOH was formed as an intermediate under autogenous pressure generated inside the autoclave, which was subsequently reduced to *Mn*_3_*O*_4_ by formic acid generated from the hydrolysis and oxidation of hexamethylenetetramine. Similarly, Jiang et al. [46] used a hydrothermal-assisted procedure, employing ethylenediaminetetraacetic acid disodium salt as the reducing agent. While these studies provide useful insight into the transformation of manganese precursors under hydrothermal conditions, their systems rely on synthetic chemical reductants and differ significantly from the green chemistry approach used in our work.

Although the specific polyphenolic compounds in EET-50 were not identified in this study, literature reports on Carménère grape pomace have described the presence of flavonoids (e.g., quercetin, kaempferol, rutin), flavan-3-ols (e.g., epicatechin, procyanidins), anthocyanins, and phenolic acids such as gallic, caffeic, and chlorogenic acids [32]. These compounds possess hydroxyl and carbonyl functional groups that can act as electron donors, facilitating the reduction of Mn^7+^ from KMnO_4_ to lower-valence manganese species under hydrothermal conditions. We hypothesize that this redox activity, combined with potential interactions between polyphenols and crystal surfaces, may direct the anisotropic growth of *Mn*_3_*O*_4_ nanocrystals, favoring the formation of octahedral morphologies.

A similar synthesis mechanism was reported by Oliveira da Silva et al. [44], who used acerola leaf extract to reduce permanganate ions through the action of compounds such as rutin, caffeic acid, ellagic acid, and ascorbic acid. In that study, the authors proposed a multi-step pathway involving (i) the reduction of Mn^7+^ to Mn^0^ by phytochemicals via electron donation and keto-enol transformation, (ii) the oxidation of reduced Mn species and nucleation of MnO_2_, and (iii) growth and stabilization of nanostructures through electrostatic interactions and capping by phytochemicals. While the phytochemical composition of EET-50 differs, its high flavonoid and anthocyanin content likely provides a comparable redox and stabilizing environment conducive to the controlled formation of *Mn*_3_*O*_4_ nano-octahedra. The appropriate selection of the KMnO_4_/EET-50 ratio and reaction time plays a crucial role in influencing the transformation of the manganese source intermediate and the oxidation of polyphenols.

#### 3.2.2. X-Ray Diffraction

**Figure 3:**
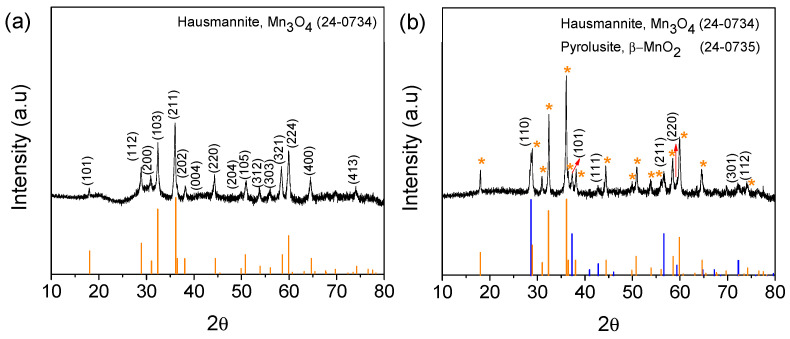
XRD patterns for samples prepared with a mass ratio of 3:1 KMnO_4_: EET-50 at (**a**) 3 and (**b**) 12 h of reaction time. Orange and blue patterns were associated with hausmannite and pyrolusite, respectively. The orange asterisks were added as a visual reference to validate the hausmannite signals shown in the graph.

The X-ray diffraction (XRD) patterns of the samples with the most representative results from the previously discussed morphology tests are presented in this section. In Figure 3, samples with reaction times of 3 and 12 h are shown.

The diffraction peaks in Figure 3a can be indexed exclusively as *Mn*_3_*O*_4_ (hausmannite), while those in Figure 3b correspond to a mixture of body-centered tetragonal *Mn*_3_*O*_4_ (hausmannite) and tetragonal β-MnO_2_ (pyrolusite), according to JCPDS cards 24-0734 and 24-0735, respectively. In Figure 3b, only Miller indices corresponding to β-MnO_2_ were labelled, while all peaks associated with hausmannite are marked with an orange asterisk. Additionally, for both diffractograms, peak positions and their relative intensities are displayed at the bottom of each graph. For further clarity, Table S1 in the Supplementary Information provides a detailed list of each peak position.

These results are consistent with the morphological observations obtained via SEM, where the first sample (3 h) consists exclusively of octahedral structures, while the second sample (12 h) exhibits a mixture of octahedral crystals and bar-like structures. This suggests that shorter reaction times favor the formation of octahedral structures, whereas longer reaction times promote structural elongation, leading to the formation of a mixture of nanorods and nano-octahedra. No characteristic peaks of impurities were detected.

Figure S3 in the Supplementary Information presents the Raman spectra for both *Mn*_3_*O*_4_ and *Mn*_3_*O*_4_/β-MnO_2_ mixtures. A prominent signal at 649.96 cm^−1^ corresponds to the A_1g_ mode of the spinel structure, associated with the Mn-O vibration of tetrahedral Mn^+2^ in *Mn*_3_*O*_4_. Additionally, peaks at 312.52 and 353.65 cm^−1^ were attributed to the vibrations of oxygen atoms in the octahedral and tetrahedral coordination, respectively, corresponding to the β-MnO_2_ [47]. Additionally, it has been reported that the peaks 312.52 and 353.65 cm^−1^ correspond to the formation of Mn_2_O_3_ and *Mn*_3_*O*_4_ caused by the incident laser energy [48]. The Raman results are consistent with XRD findings, confirming that the sample obtained after a shorter reaction time consists of a single phase, while the sample with the longer reaction time contains two phases.

#### 3.2.3. Electrochemical Measurements

Figure S4a,b (in Supplementary Material) shows the cyclic voltammograms of electrodes modified with *Mn*_3_*O*_4_ and *Mn*_3_*O*_4_/β-MnO_2_ at different scan rates. A broad peak near 0.250 V in the negative potential direction was observed for both electrodes, corresponding to the reduction of Mn^+3^ to Mn^+2^. In the potential range of 0.580 to 0.800 V, the anodic current for *Mn*_3_*O*_4_ was significantly broader than that for the *Mn*_3_*O*_4_/β-MnO_2_ mixture. This behavior was attributed to the fact that the Mn^+2^ → *Mn*_3_*O*_4_ reaction occurs at a lower potential than the Mn^+2^ → MnO_2_ reaction [49].

Although both *Mn*_3_*O*_4_ and β-MnO_2_ are theoretically partially capacitive, they also exhibit redox behavior. Notably, *Mn*_3_*O*_4_ displays more defined redox peaks than those corresponding to β-MnO_2_ [50,51]. However, in the present study, the cyclic voltammograms did not show clearly distinguishable peaks; thus, the characteristic peaks associated with the Mn^+2^ → Mn^+3^ and Mn^+3^ → Mn^+4^ transitions in *Mn*_3_*O*_4_ were not observed. Nonetheless, a small peak near 0.350 V, associated with the first oxidation of Mn^+2^ → Mn^+3^, can be identified in the *Mn*_3_*O*_4_ voltammogram (Figure S4a), while it is absent in the *Mn*_3_*O*_4_/β-MnO_2_ mixture (Figure S4b). As for the second oxidation process, typically reported between 0.6 and 0.7 V vs. Ag/AgCl, only a broad current response was observed in this work, more pronounced than that of β-MnO_2_ (Figure S4b).

The specific capacitances were calculated from Equation (4) and plotted as a function of the scan rate (Figure S4c). Maximum capacitance values of 77.4 and 25.7 F/g were obtained at 0.01 V/s for *Mn*_3_*O*_4_ and *Mn*_3_*O*_4_/β-MnO_2_, respectively. At a scan rate of 0.150 V/s, a decrease in retention was observed for both materials, obtaining 21.58 and 22.2% of the maximum charge, respectively. This decrease in capacitance is attributed to the increase in scan rate, where ion transport to the electrode/solution interface becomes limited by the migration of ions from the bulk solution [36].

Furthermore, the *Mn*_3_*O*_4_ modified electrode exhibits a higher specific capacitance than the *Mn*_3_*O*_4_/β-MnO_2_-modified electrode, which could be attributed to the charging mechanisms of these materials with different crystal structures (Figure S4d).

**Figure 4:**
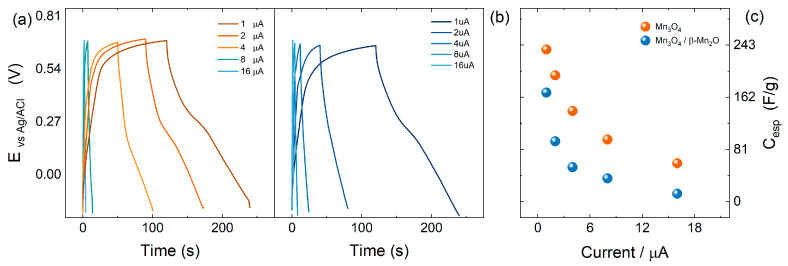
Galvanostatic charge/discharge curves of (**a**) *Mn*_3_*O*_4_ and (**b**) *Mn*_3_*O*_4_/β-MnO_2_, in 1 mol/L Na_2_SO_4_ at different current densities. (**c**) Specific capacitance of the modified electrode at different current densities.

Figure 4a,b display GCD curves corresponding to the modified *Mn*_3_*O*_4_ and *Mn*_3_*O*_4_/β-MnO_2_ electrodes at different current densities between −0.2 to 0.6 V. Specific capacitances were calculated using Equation (5). At lower current densities (1 mA/g), capacitance values of 236.27 F/g and 169.44 F/g were obtained for *Mn*_3_*O*_4_ and *Mn*_3_*O*_4_/β-MnO_2_, respectively, representing a 28.3% reduction for a mixture of manganese oxides. At higher current densities (16 mA/g), this difference in specific capacitance becomes more pronounced, reaching 79.54%, with values of 59.47 F/g and 12.17 F/g for *Mn*_3_*O*_4_ and *Mn*_3_*O*_4_/β-MnO_2_, respectively.

These results highlight the superior electrochemical performance of *Mn*_3_*O*_4_ compared with MnO_2_, which may be attributed to the charge–discharge mechanisms. For *Mn*_3_*O*_4_, a redox-based mechanism has been described where the Mn^2+^ species were oxidized to Mn^3+^ during the first cycle. This process allows the insertion of Na^+^ ions, promoting a phase change as outlined in Equation (6) [13].

(6) $${Mn}_{3}O_{4}\to {Na}_{\gamma }MnO_{\chi }\times \eta H_{2}O$$

**Figure 5:**
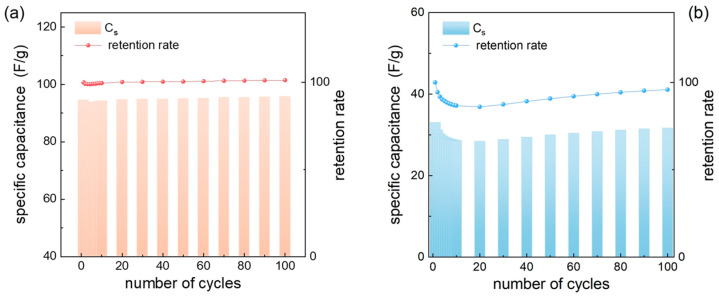
Charge–discharge of the modified electrode at a constant current density of 8 mA/g of (**a**) *Mn*_3_*O*_4_ and (**b**) *Mn*_3_*O*_4_/β-MnO_2_.

For β-MnO_2_, several charge storage mechanisms have been described in the literature. Among them, the tunnel storage mechanism, in which the Na^+^ ion migrates into the beta phase (Figure 5b), is considered the most promising option, with a reported specific capacitance of approximately 9 F/g [52,53]. However, the charging mechanism for *Mn*_3_*O*_4_ remains not fully elucidated. Therefore, it can be proposed that the difference in specific capacitance between these materials may be attributed to variations in their specific surface areas [54].

Figure 5 shows the GCD curves at 8 mA/g for *Mn*_3_*O*_4_ and *Mn*_3_*O*_4_/β-MnO_2_ modified electrodes over 100 cycles. The *Mn*_3_*O*_4_-modified electrodes exhibit superior stability, with 99.98% retention. In contrast, the *Mn*_3_*O*_4_/β-MnO_2_ modified electrode retains 95.91%. However, it is also important to mention that a rapid decay in the charge retention is observed in both cases, where the *Mn*_3_*O*_4_ pair recovers quickly during the first cycles, whereas the *Mn*_3_*O*_4_/β-MnO_2_ mixture takes six times longer to recover its initial charge. This behavior is attributed to the presence of β-MnO_2_, which exhibits slower material activation, leading to delayed ion penetration compared to *Mn*_3_*O*_4_ [55].

**Figure 6:**
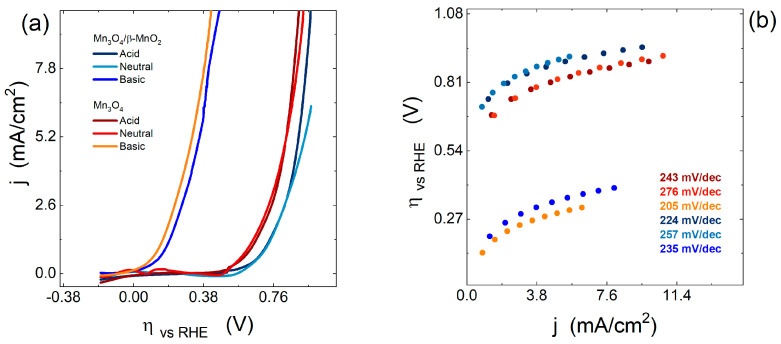
Oxygen evolution reaction of modified electrode *Mn*_3_*O*_4_ and *Mn*_3_*O*_4_/β-MnO_2_ obtained in different media (**a**) LSV curves, (**b**) Tafel plots.

In parallel, the catalytic properties against the OER were evaluated for the *Mn*_3_*O*_4_ and *Mn*_3_*O*_4_/β-MnO_2_ modified electrodes in different media. Figure 6a shows LVS obtained at 0.002 V/s, where the *Mn*_3_*O*_4_-modified electrode exhibits a lower overpotential at 10 mA/cm^2^ (η_10_), with values of 0.430, 0.930, and 0.900 V vs. OER in basic, neutral, and acidic media, respectively. In contrast, *Mn*_3_*O*_4_/β-MnO_2_ modified electrode shows η_10_ values of 0.490 and 0.970 V vs. RHE in basic and acidic media, while in neutral media, it reaches current densities of 6.4 mA/cm^2^ with an overpotential of 0.970 V vs. RHE.

The small difference in overpotential between the two electrodes can be attributed to the morphology of the nanostructures: nano-octahedra for *Mn*_3_*O*_4_ and nanorods for β-MnO_2_, where the surface area would play a predominant role. Additionally, the lower overpotential values obtained in the basic media for both electrodes are consistent with the mechanism reported in the literature [56].

On the other hand, the OER kinetics were further analyzed using Tafel slopes obtained for the *Mn*_3_*O*_4_ and *Mn*_3_*O*_4_/β-MnO_2_ modified electrodes in basic, neutral, and acidic media (Figure 6b). In all cases, high Tafel slope values were observed, which is directly related to the four-electron transfer for this type of reaction [57]. Despite this, the *Mn*_3_*O*_4_-modified electrode exhibits a lower Tafel slope compared to the other systems, which added to a lower overpotential, would indicate better electrocatalytic properties in comparison with *Mn*_3_*O*_4_/β-MnO_2_-modified electrode in all the environments studied.

It is important to mention that the commercially available IrO_2_-based electrocatalyst has overpotentials of 0.327 ± 0.010 V at 10 mA/cm^2^, a performance attributed to the intrinsic catalytic capacity of Ir [58]. However, *Mn*_3_*O*_4_ could be an interesting support for this type of material, further decreasing the overpotential for OER.

In addition, Table 2 presents data reported in the literature on the capacitive and catalytic properties of *Mn*_3_*O*_4_, synthesized via different methods. Regarding the storage properties of *Mn*_3_*O*_4_, lower capacitance values were observed for those obtained by the hydrothermal route. This behavior can be attributed to the different morphologies reported (nanoparticles and mixed with nanorods and nanoparticles), but this higher specific capacitance would be mainly related to the size of the nanostructure, being 67 and 350% smaller than those reported in literature [59,60].

Although the capacitance values obtained via electrochemical and co-precipitation methods were between 1.5 and 2 times higher than those reported in the present study, this is likely due to their smaller particle sizes, which in both cases are close to 100 nm- about 50% smaller [59]. It is also important to mention that in all the cases presented in Table 2, the electrochemical performance was enhanced by incorporating 10% carbon black, which improves electrode conductivity of the electrode and facilitates more efficient Na^+^ intercalation.

On the other hand, the catalytic properties of *Mn*_3_*O*_4_ obtained in the present work show an interesting behavior from the thermodynamic standpoint, consistently showing lower overpotentials for OER compared to those described in literature (Table 2). This improvement is attributed to the morphology of the nano-octahedra, which was more reactive than those described in the literature [61,62,63].

**Table 2:** Comparative table of different *Mn*_3_*O*_4_ syntheses.

Morphology	Specific Capacitance	h/V	Reference
Nanoparticles ^a^	198 F/g at 0.5 mA/cm^2^	---	[64]
Nanorods and nanoparticles ^a^	233.41 F/g at 0.5 A/g	---	[60]
Nanoparticles embedded in nanorods ^c^	499.6 F/g at 1 mV/s	---	[65]
Nanoparticles ^d^	---	0.450 at 1 mA/cm^2^	[63]
*Mn*_3_*O*_4_ ^b^	---	0.570 at 10 mA/cm^2^	[62]
*Mn*_3_*O*_4_ ^e^	---	0.582 at 1,4 mA/cm^2^	[61]
Nano-octahedra ^a^	236.27 F/g at 1 mA/g	0.430 at 10 mA/cm^2^	This work
Nano-octahedra and nanorods β-MnO_2_ ^a^	169.4 F/g at 1 mA/g	0.490 at 10 mA/cm^2^	This work

Method: ^a^ hydrothermal, ^b^ electrochemical, ^c^ co-precipitation, ^d^ hot injection, ^e^ microwave.

Finally, it is important to discuss the relationship between nano-octahedral morphology and its storage and catalytic properties. Huang et al. [11] proposed two main strategies to enhance the conductivity and electrochemical performance of *Mn*_3_*O*_4_-based materials: (i) the development of *Mn*_3_*O*_4_ composites or hybrid structures incorporating components that improve electrode conductivity, and (ii) the design of nanostructures with a high density of exposed electroactive sites to promote more efficient electrochemical reactions. In this work, the second strategy was implemented by promoting the formation of well-defined *Mn*_3_*O*_4_ nano-octahedra with high-energy exposed facets, in contrast to a mixed crystalline phase. Furthermore, [66] highlighted that smaller nano-octahedral crystals offer a greater number of active sites in direct contact with the electrolyte and facilitate shorter pathways for charged transport. In this investigation, it was observed that the sample composed exclusively of nano-octahedra exhibited particle sizes in the range of 100–170 nm, while in the mixed-phase sample, the nano-octahedra were slightly larger, ranging from 150–250 nm. This morphological difference may account for the enhanced electrochemical performance observed in the single crystal sample, underscoring the crucial role of crystal size and shape in governing electrochemical behavior.

## 4. Discussion

The green hydrothermal synthesis method developed in this work offers significant potential for energy storage and catalytic applications. The *Mn*_3_*O*_4_ nano-octahedra, with a high specific capacitance of 236.27 F/g at 1 mA/g and exceptional cycling stability (99.98% retention after 100 cycles at 8 mA/g), are promising candidates for use in supercapacitors. Additionally, their catalytic performance in the OER, with an overpotential of 1.66 V vs. RHE and a Tafel slope of 205 mV/dec, makes them suitable for water-splitting systems.

The use of EET-50, a biomolecule-rich extract from Carménère pomace, underscores the potential of agricultural by-products as sustainable resources for advanced material synthesis, aligning with green chemistry principles.

Future research could focus on optimizing synthesis parameters—such as reaction time, temperature, and precursor ratios—to achieve better control of morphology and phase composition. Exploring hybrid systems, such as combining these materials with conductive polymers or carbon-based structures, could further enhance their electrochemical properties. Additionally, their application in other catalytic processes, such as CO_2_ reduction or pollutant degradation, should be investigated. Scaling up the synthesis process while maintaining its eco-friendly nature will be crucial for practical implementation. Furthermore, a deeper understanding of the role of polyphenols in nanoparticle formation is needed. Beyond their reducing capacity, it is hypothesized that these biomolecules may act as capping agents during the growth phase, influencing the stabilization and morphology of the nanostructures. Investigating the molecular interactions between secondary metabolites and developing nanoparticles will be essential to refining the synthesis mechanism and enhancing control over the resulting materials.

This work highlights the potential of green synthesis approaches for developing high-performance materials, paving the way for sustainable solutions in energy storage and catalysis.

## 5. Conclusions

The green hydrothermal synthesis method was successfully used to produce *Mn*_3_*O*_4_ and *Mn*_3_*O*_4_/β-MnO_2_ nanostructures using EET-50, a biomolecule-rich extract derived from a Carménère pomace. Polyphenols, flavonoids, and anthocyanins in EET-50 functioned as effective reducing agents, enabling nanostructure formation. SEM analysis revealed that a KMnO_4_/EET-50 mass ratio of 3:1 led to the development of well-defined nano-octahedra combined with rods, while shorter reaction times favored the formation of isolated nano-octahedra with dimensions ranging between 100 nm and 170 nm. XRD and Raman confirmed the presence of mixed-phase *Mn*_3_*O*_4_/β-MnO_2_ and *Mn*_3_*O*_4_, respectively. Electrochemical tests showed that *Mn*_3_*O*_4_ nano-octahedra achieved a specific capacitance of 236.27 F/g at 1 mA/g, exceeding the mixed-phase material by 28.3% with exceptional capacitance retention of 99.98% after 100 cycles. Additionally, they exhibited superior OER performances in alkaline media. These results highlight the potential of green synthesis using sustainable by-products for developing high-performance nanomaterials for energy storage and catalysis.

From a practical perspective, this synthesis approach offers both environmental and economic benefits by valorizing grape pomace, an abundant and low-cost by-product of the Chilean wine industry, in line with circular economy principles. To address reproducibility challenges typically associated with natural extracts, we recorded the harvest year and geographic origin of the raw material and chemically standardized the extract by determining total phenolic, flavonoid, and anthocyanin content, along with antioxidant activity. Furthermore, the extraction conditions used were simple, robust, and compatible with industrial processes, supporting potential scale-up. When compared to other *Mn*_3_*O*_4_-based systems, the materials obtained in this work demonstrate competitive—often superior—electrochemical performance, reinforcing the relevance of green synthesis as a viable and sustainable alternative to conventional chemical methods.

## Figures and Tables

**Table 1:** Phenolic content and in vitro antioxidant activity by ferric-reducing antioxidant power (FRAP) and DPPH radical method of the ethanolic extract of Carménère grape.

Yield (%)	Phenolic Content			Antioxidant Activity	
	TPC ^1^ (mgGAE/dw)	TFC ^2^ (mgQE/gdw)	TMA ^3^ (mgCE/gdw)	FRAP ^4^ (mmolTE/dw)	DPPH, IC_50_^5^ (µg/mL)
7.8 ± 0.1	149.9 ± 0.4	12.1 ± 0.9	12.1 ± 0.4	1.59 ± 0.04	17.96 ± 0.87

^1^ TPC: total phenolic content (mg gallic acid equivalent/g dry weight extract); ^2^ TFC: total flavonoid content (mg quercetin equivalent/g dry weight extract^)^; ^3^ TMA: total monomeric anthocyanin content, (mg cyanidin-3-glucoside equivalent/g dry weight extract); ^4^ FRAP: ferric-reducing antioxidant power (mmol Trolox equivalent/g dry weight extract); ^5^ DPPH: inhibitory concentration 50 (µg/mL).

## Data Availability

Restrictions apply to the datasets presented in this article because they are subject to confidentiality agreements. Requests to access the datasets should be directed to the corresponding authors.

## Supplementary Materials

The following supporting information can be downloaded at: https://www.mdpi.com/article/10.3390/nano15161282/s1, Figure S1: TEM images of the sample with KMnO_4_/EET50 3:1 at 12 h of reaction time (reaction temperature: 180 °C); Figure S2: TEM images of the sample with KMnO_4_/EET50 3:1 at 80 °C of reaction temperature (reaction time: 12 h), Figure S3: Raman spectra of the samples were obtained from a 3:1 KMnO_4_/EET50 mass ratio at (orange) 3 and (blue) 12 h, Figure S4: Cyclic voltammograms in different scan rates of the modified electrodes (a) Mn_3_O_4_ and (b) Mn_3_O_4_/β-MnO_2_, in 1 mol/L Na_2_SO_4_. (c) Specific capacitance of the modified electrode is a function of the scan rate (d), crystalline structures of Mn_3_O_4_ and β-MnO_2_, Table S1: Angle, d-spacing, and Miller indexes for the samples obtained with 3 and 12 h of reaction time.

## Abbreviations

The following abbreviations are used in this manuscript:

EET-50	50% *v*/*v* hydroalcoholic extract from grape pomace
TEM	Transmission electron microscopy
SEM	Scanning electron microscopy
XRD	X-ray diffraction
ORR	Oxygen reduction reaction
OER	Oxygen evolution reaction
RHE	Reversible hydrogen electrode
TPC	Total phenolic content
TFC	Total flavonoid content
TMA	Total monomeric anthocyanin content
MW	Molecular weight
DF	Dilution factor
ε	Molar absorptivity
FRAP	Ferritic reducing antioxidant power
DPPH	2,2-diphenyl-βpicrylhydrazyl
TPTZ	Ferric-2,4,6,tripyridyl-s-triazine
IC50	Inhibitory concentration μg·mL^−1^
GC	Glassy carbon electrode
CDG	Charge–discharge process at constant current
LSV	Linear sweep voltammetry
SET	Single-electron transfer hydrogen
HAT	Hydrogen atom transfer
