# Supplementary material for: Green Hydrothermal Synthesis of Mn3O4 Nano-Octahedra Using Carménère Grape Pomace Extract and Evaluation of Their Properties for Energy Storage and Electrocatalysis
Source: Nanomaterials (Basel). 2025 Aug 20;15(16):1282. doi: 10.3390/nano15161282 (PMC12389097; doi:10.3390/nano15161282)
Supplement: Supplementary file 1 [file nanomaterials-15-01282-s001.zip › nanomaterials-3778986-supplementary.pdf]

Supplementary material

Green Hydrothermal Synthesis of Mn_3O_4 Nano-octahedra using Carménère Grape Pomace Extract and Evaluation of their Properties for Energy Storage and Electrocatalysis

Javier Lorca-Ponce[1], Paula Valenzuela-Bustamante[1], Paula Cornejo Retamales[1], Nicolás Nolan Mella[1], Valentina Cavieres Ríos[1], M.J. Pérez Velez[2], A.M.R. Ramírez[2*] and Leslie Diaz Jalaff[1*]

[1]Centro de Excelencia en Materiales Avanzados - Nanotecnología, LEITAT Chile, Santiago 7500724, Chile

[2]Departamento de Química, Facultad de Ciencias, Universidad de Chile, Santiago 7800003, Chile

E-mail: andrramirez@uchile.cl, ldiaz@leitat.cl

The supporting information contains:

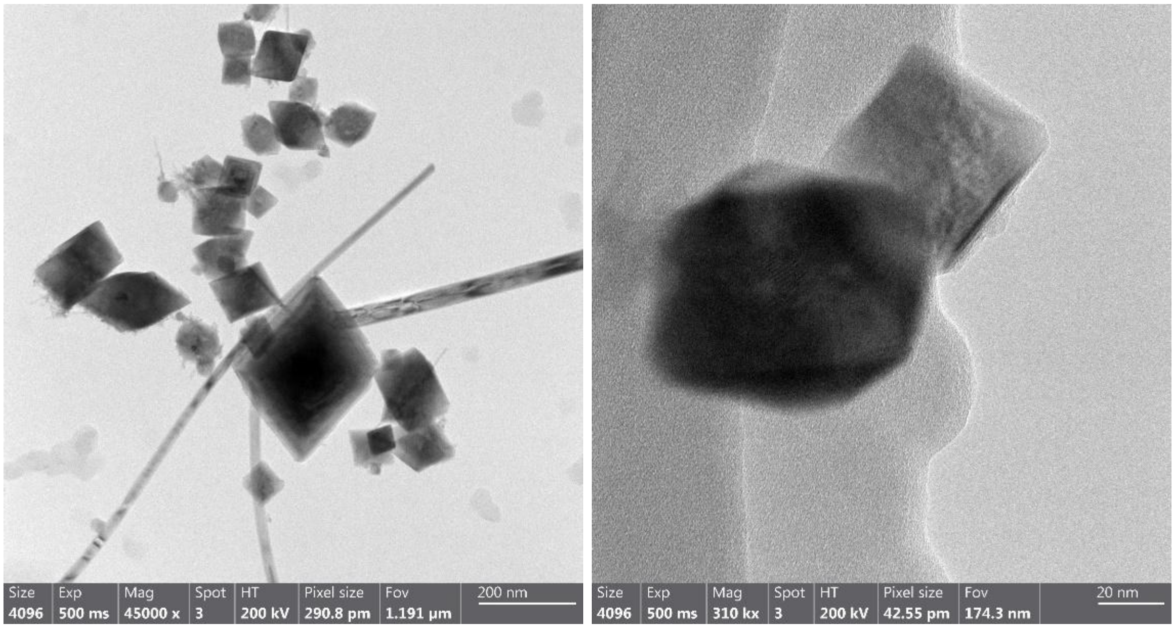

Figure S1. TEM images of the sample with $KMnO_4$: EET50 3:1 at 12 h of reaction time (reaction temperature: 180°C)

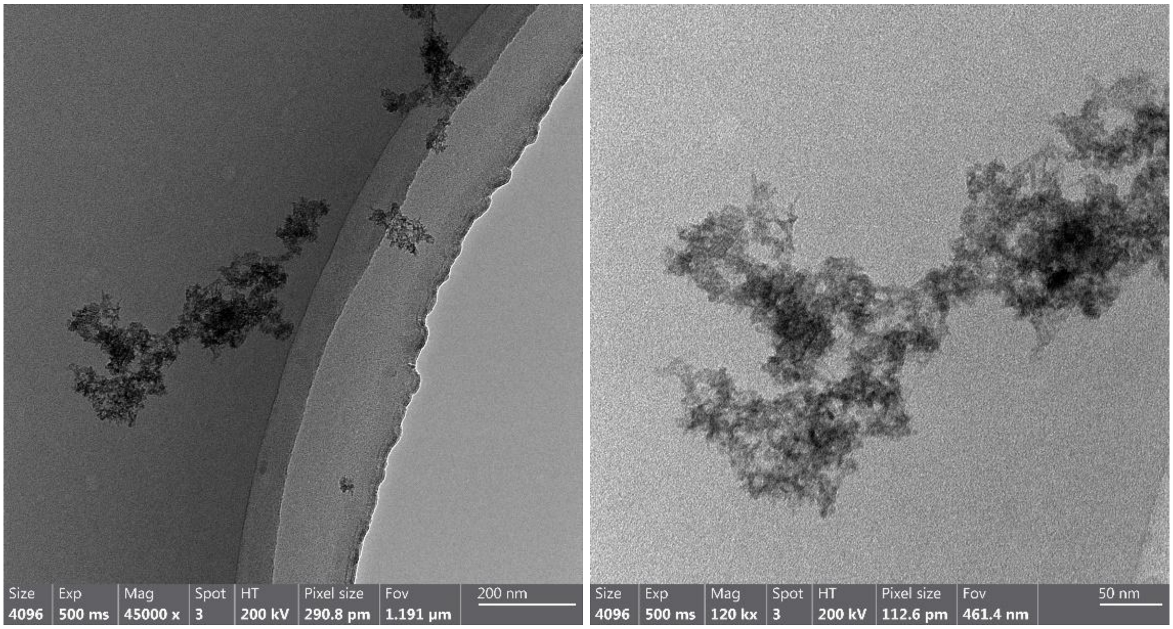

Figure S2. TEM images of the sample with $KMnO_4$: EET50 3:1 at 80°C of reaction temperature (reaction time: 12 h)

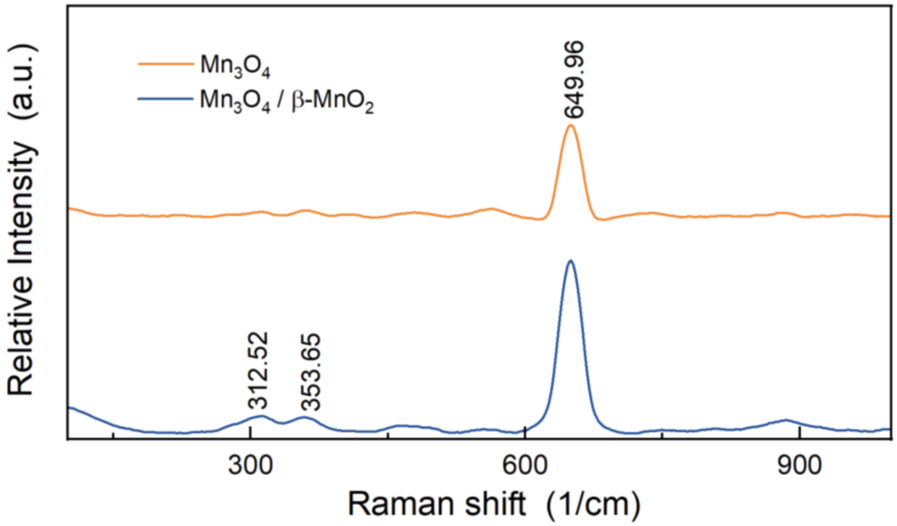

Figure S3. Raman spectra of the samples were obtained from a 3:1 $KMnO_4$: EET50 mass ratio at (orange) 3 and (blue) 12 h

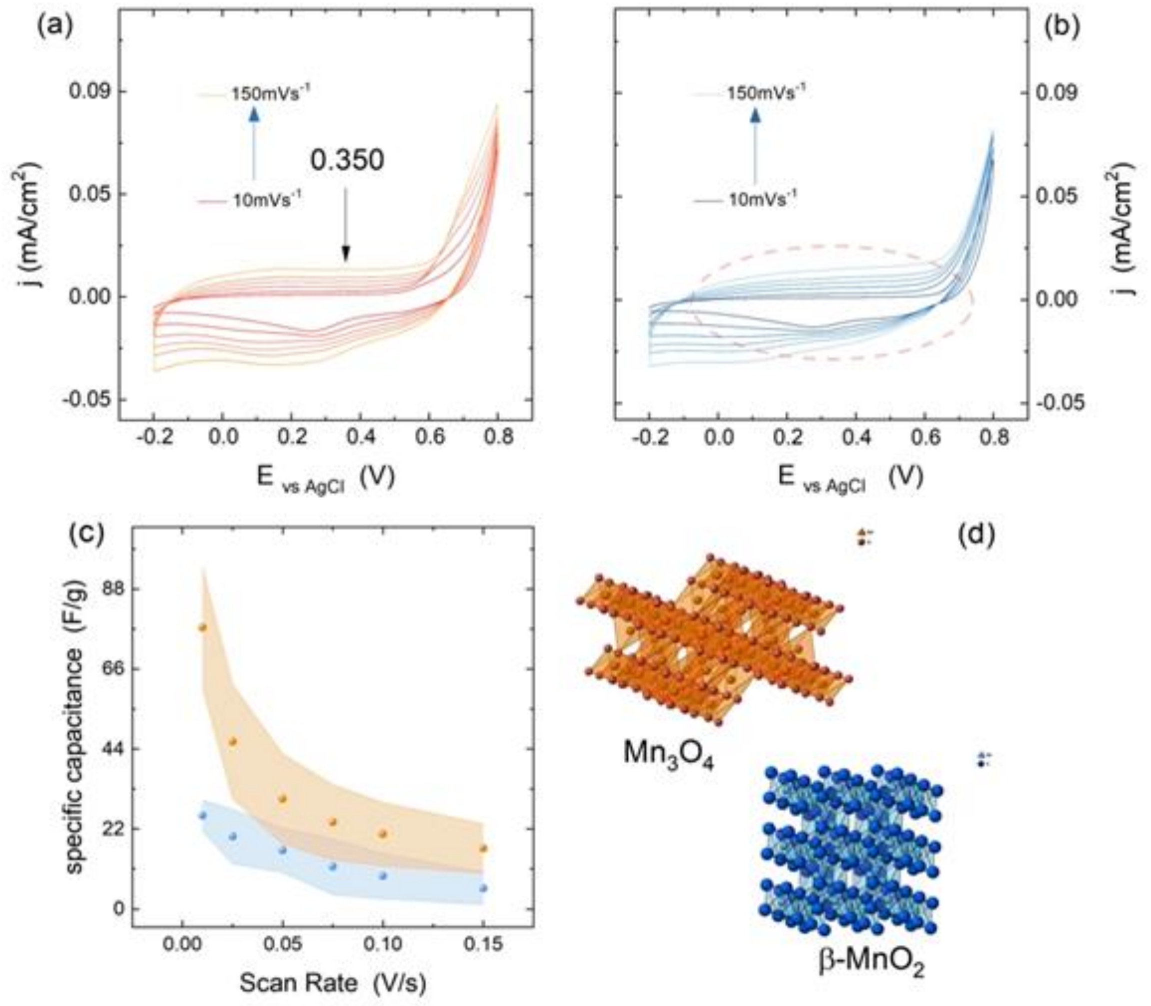

Figure S4. Cyclic voltammograms in different scan rates of the modified electrodes (a) Mn_3O_4 and (b) Mn_3O_4/β-MnO_2, in 1 mol/L Na_2SO_4. (c) Specific capacitance of the modified electrode is a function of the scan rate (d) crystalline structures of Mn_3O_4 and β-MnO_2

Table S1. Angle, d-spacing and Miller indexes for the samples obtained with 3 and 12 hours of reaction time

**3 h**			**12 h**					
**Mn_3O_4**			**Mn_3O_4**			**β-MnO_2**		
**2θ**	**d (Å)**	**(hkl)**	**2θ**	**d (Å)**	**(hkl)**	**2θ**	**d (Å)**	**(hkl)**
17.90	4.951	101	18.02	4.918	101	28.66	3,112	110
28.82	3.095	112	28.94	3.083	112	3734	2,406	101
30.89	2.892	200	30.97	2.885	200	42.83	2,110	111
32.41	2.760	103	32.43	2.758	103	56.50	1,627	211
36.04	2.490	211	36.08	2.487	211	59.28	1,557	220
36.32	2.471	202	36.45	2.463	202	72.13	1,308	301
38.01	2.365	004	38.09	2.360	004	72.62	1,301	112
44.37	2.040	220	44.37	2.040	220			
49.91	1.826	204	49.89	1.826	204			
50.90	1.792	105	50.90	1.792	105			
53.60	1.708	312	53.68	1.706	312			
55.85	1.645	303	56.01	1.640	303			
58.46	1.577	321	58.44	1.578	321			
59.90	1.543	224	59.90	1.543	224			
64.47	1.444	400	64.49	1.444	400			
73.93	1.281	413	73.93	1.281	413			
